# The unique characteristics of “families of choice” and families of origin of older LGBTQ + adults: are they related to mental health?

**DOI:** 10.1007/s00127-025-02919-y

**Published:** 2025-05-14

**Authors:** Ella Cohn-Schwartz, Sigal Gooldin, Lian Meiry, Robert Heidemann, Yaacov G. Bachner

**Affiliations:** 1 Gerontology Program, Department of Epidemiology, Biostatistics, and Community Health Sciences, School of Public Health, Ben-Gurion University of the Negev, Beer-Sheva, Israel; 2 The Israeli Institute for Gender and LGBTQ Studies, Tel Aviv, Israel; 3 Department of Physiotherapy, Ben-Gurion University, Be’er Sheva, Israel; 4 Department of Social Sciences, TU Dortmund University, Dortmund, Germany

**Keywords:** LGBT, Social relationships, Friends, Family, Depression, Well-being

## Abstract

**Purpose:**

This study joins a growing body of research on the different types of families of aging individuals belonging to sexual and gender minorities. We explore the characteristics of “families of choice” (who are close enough to be considered as family) and families of origin of LGBTQ + older adults and their associations with mental health.

**Methods:**

Data for this study were collected via an online survey with self-identified lesbian, gay, bi-sexual, transgender, queer and others (LGBTQ +) adults aged 50 + (*n* = 432). Participants were asked about characteristics of the relationships with their families of choice and their families of origin, depressive symptoms and well-being.

**Results:**

The results indicated that most participants had a family of choice, numbering five people on average. They also reported having about four close family of origin members. Several differences emerged when comparing the two types of families: The relationships with families of origin were more stable, while families of choice were more committed, more likely to accept participants' sexual orientation and the relationship with members of families of choice had fewer negative aspects. Regression analyses showed that individuals had better mental health if they had more close family of origin members, more family of choice members and a partner, if the relationship with their families of choice were more stable and less negative.

**Conclusion:**

These findings shed light on the unique sources of support among LGBTQ + older adults and their associations with mental health.

## Introduction

The world population, including a diverse population of sexual and gender minorities, is rapidly aging. Older lesbian, gay, bisexual, transgender (Transgender individuals are those whose gender identity differs from the sex they were assigned at birth), queer and more (LGBTQ +^1^) individuals represent an understudied group within the larger aging population, with distinct characteristics and needs that should be understood and addressed [1]. Estimates indicate that LGBTQ + adults comprise between 5.5 and 9.6% of the general population [2–4]. LGBTQ + older adults, as compared to older heterosexual cis-gender adults (people whose gender identity corresponds with the sex they were assigned at birth), face an increased risk of mental health issues, such as depression and diminished well-being, while also being at greater risk of social isolation [4, 5]. Many have no children and are estranged from their biological families, leading to unique social environments (e.g., families of choice) that differ from those of older heterosexual adults.

Social relationships are integral to the mental health of heterosexual and LGBTQ + older adults alike [6–8]. However, compared to heterosexual cis people, social relationships might be of increased importance for LGBTQ + older adults, as they could need more support in the face of discrimination. In addition to experiences of discrimination at the individual level, sexual and gender minorities are also affected by structural stigmatisation [9] and LGBTQ + older adults are more likely to experience discrimination when dealing with authorities, healthcare providers and other institutions [10]. These discriminatory experiences and the lack of trust among older LGBTQ + adults are likely to be all the greater, as they grew up at a time when sexual and gender minorities were criminalized, pathologized and excluded (for an overview of the legal framework in Israel, see [11]). In terms of a life course perspective, however, discriminatory experiences with institutions in earlier life can lead to avoidance of services in the present for fear of encountering rejection again and not being taken seriously [12]. Due to these dynamics, the importance of social relationships could be particularly high for the mental health and well-being of older LGBTQ + people. However, the effects of the older LGBTQ + population’s unique social relationships, such as their families of choice, are not yet well understood. Therefore, this study aims to better understand these unique social relationships and their association with the mental health of LGBTQ + adults in later life.

### Social relationships of LGBTQ + adults

LGBTQ+ older adults often have social relationships that differ from those of heterosexual older adults. One aspect in need of more rigorous investigation is ties with the family. In contrast to the centrality of family members in the lives of heterosexual older adults, LGBTQ + older adults often have limited ties with their families of origin, and many do not have children of their own and/or live alone [13]. Contact with families of origin might be hampered by a lack of disclosure or acceptance of the sexual identity of LGBTQ + individuals [14]. With more limited ties, many LGBTQ + older adults forge “families of choice”, a term that refers to social ties with others who are not biologically related to them, such as friends and partners [15–19]. They are considered to be people to whom one is emotionally close and considers “family” [20]. The term “families of choice” was introduced by Kath Weston in 1997. She used it during the HIV/AIDS crisis when traditional family ties were strained or failed, to highlight the importance of non-biological relationships, such as friendships, which were the main providers of care and support [21]. Research supports the assertion that the social relationships of LGBTQ + older adults, more so than those of heterosexual older adults, largely include partners and friends [13, 14, 22]. The concept of families of choice is prominently used in LGBTQ + communities, especially among people who experience rejection from their families of origin. These family of choice members are described as being understanding and loving, and as helping LGBTQ + individuals navigate the challenges of life [23].

Although important in the LGBTQ + literature and everyday life, families of choice are not distinguished from other friends or explicitly defined in most quantitative research regarding LGBTQ + older adults (e.g., [13, 14]). An example of their centrality was illustrated by the finding that 64% of LGBTQ + adults aged 45–64 had a “chosen family” [20]. Thus, families of choice seem to be prevalent in the lives of older LGBTQ + adults and should receive specific attention in the scientific literature. It is especially important to deepen our understanding of the connection between families of choice and mental health, as LGBTQ + older adults may face heightened risks to their mental well-being.

### Mental health of LGBTQ+ older adults

Compared to heterosexual older adults, research suggests that older LGBTQ + adults experience considerably more depressive episodes relative to the general population, while reporting fewer feelings of happiness [5, 24–26]. For example, a U.S. study found that 31% of older LGBTQ + adults were depressed [27], more than twice the percentage of heterosexual older Americans [28]. As they grow older, LGBTQ + adults can experience worsening mental health since they encounter worsening homophobia and ageism, and believe that their sexual orientation will negatively affect them as they grow older [29].

### Mental health and social relationships

Social relationships can foster positive mental health among older LGBTQ + adults [30, 31]. Indeed, larger social networks and more social support are associated with lower depression among LGBTQ + older adults [32, 33], and with higher quality of life [34]. Moreover, social support from friends, but not from family, has been found to be related to lower depression among this group [35]. However, studies of the social relationships among LGBTQ + older adults did not fully consider the unique characteristics of their social relationships in relation to mental health: They did not specifically focus on families of choice (more often examining “friends” in general), nor did they examine the effects of the unique characteristics of families of choice.

The Health Equity Promotion Model (HEPM; [36]) details which structural, individual and social components are decisive for the health of sexual minorities. A differentiated view and consideration of intersections (i.e. consideration of the interweaving of characteristics such as ethnicity, class and gender) results in a multi-layered view of health differences within the group of sexual minorities and clarifies hindering and favourable factors for good health and well-being. In doing so, it identifies meaningful social relationships as fostering resilience [36, 37]. However, the HEPM does not offer more specific predictions regarding different attributes of LGBTQ + social relationships. The model conceptualized social resources in general as health promoting, and research based on this model examined social resources broadly, for example using general measures of social support or a measure of being partnered [1, 34, 36]. To better understand the role of LGBTQ + older adults’ social relationships, the current study will incorporate the convoy model of social relations [38]. This theoretical model conceptualizes social relationships as “social convoys”, a collection of people who surround individuals during the life course, move with them over time and are available as resources of support in times of need [39]. The convoy model maintains that social ties are instrumental for coping with the challenges of old age and theory particularly underscores the significant implications of social relationships for the psychological well-being of older adults [40]. These relationships vary in their structure (e.g., size, contact frequency) and quality (e.g., emotional support), with relationship quality often having a greater impact on mental health than relationship structure [38].

The convoy model claims that the quality of relationships, namely the emotional support they provide, is particularly relevant for mental health. However, among LGBTQ + older adults, additional aspects might be relevant, such as the commitment and stability of their social ties. Families of origin are typically characterized by obligatory commitment and stability, often reinforced by legal, social, and cultural expectations. These institutional supports and cultural expectations of family duty may lead to sustained availability of practical assistance during times of need, even in the context of strained relationships. In contrast, while families of choice can offer emotional closeness, they may face unique challenges in maintaining long-term caregiving commitments due to their informal nature and lack of legal protections [19]. Some LGBTQ + older adults, for example, may feel uncertain about whether their friends will provide care and support in the event of declining health, particularly in the absence of formalized commitments such as legal guardianship or cohabitation [41, 42]. This complexity highlights the dual role of the family of origin—while it may be a source of support in times of need, it can also be perceived negatively due to past experiences of rejection or ambivalence. Acceptance of one’s sexual identity is an additional aspect with unique implications for LGBTQ + older adults, especially among their families of origin [14, 43].

The current study will investigate the associations of families of choice, compared to those of families of origin, with mental health. The importance of families of origin in the lives of heterosexual older adults is emphasized by the convoy model, and we will build upon this assertion to examine whether families of choice play a similar role in the lives of LGBTQ + older adults. We expect that the role of families of choice in relation to mental health will be greater than the role of families of origin. This study will build upon and expand the perspective of the convoy model to examine unique aspects in the social ties of older LGBTQ+ adults which are currently not included in this model, such as issues of commitment, stability and acceptance of sexual identity. Thus, we aim to delineate a more precise account of the particular social experiences of LGBTQ + older adults and to assess them in relation to measures of mental health, namely depressive symptoms and well-being. We hypothesize that: (1) The families of choice of LGBTQ + older adults will be characterized by lower stability and commitment, higher acceptance of one's sexual orientation and less negativity, compared to the family of origin (H1). (2) LGBTQ+ older adults with better relationships (characterized by larger networks, higher commitment, stability, acceptance and low negativity), will report better mental health in terms of fewer depressive symptoms and better sense of well-being (H2). (3) The association of families of choice with mental health will be stronger compared to that of family of origin (H3).

## Methods

### Participants and procedure

We collected the data via online surveys. Due to difficulty in reaching aging sexual and gender minorities [37], an invitation to take part in the study was published on social media, through WhatsApp groups of the LGBTQ + networks and community organizations, Facebook groups and paid online advertising. The respondents were also asked to send the survey link to other potential respondents in their social circle using the “snowball” method. Participants were recruited through collaboration with the Israeli Institute for Gender and LGBTQ Studies, a community based research institute that works with local LGBTQ organizations. Inclusion criteria were self definition as LGBTQ +, age 50 and over and ability to understand the questionnaire language. The 50 + age cut-off is consistent with prior research with older sexual minority adults (e.g., [1, 6, 26]). It also reflects what is considered “older” among the various groups in the sample. For instance, some gay men may view themselves as older earlier than non-gay adults due to the emphasis on youth and masculinity in the gay community [29]. Participants signed an informed consent form at the beginning of the questionnaire, in which they were assured that their identities would be safeguarded and that the collected data would be used only for research purposes. Filling the questionnaire was anonymous. Respondents who answered the survey had the option to leave their contact information and receive a gift voucher of about 25 USD. The questionnaires were available in Hebrew, Arabic and English, with the majority (98%) being filled in Hebrew and the rest filled in English. The study was approved by the institutional review board (IRB) of Ben-Gurion University.

Overall, 492 adults who self-identify as lesbian, gay, bisexual trans or other (non-heterosexuals) filled the questionnaires. The sample was limited to those who reported having a family of choice or a partner (most partners—97%—were mentioned in the family of choice, and 7% (35) of respondents reported not having a family of choice and not having a partner). The sample was also limited to those who had full information on the study variables, thus 25 participants who did not have full information were excluded. The final study sample focused on 432 participants who had full information on the study variables. Sample size calculation was done for linear multiple regression. We have assessed the sample size required for a model with 13 variables, in which the variance explained by the full model is 0.25. Thus, considering a Type 1 error of 5% and a power of 80%, our sample size calculation yielded an *N* of 210. Therefore, our sample size (*n* = 432) should have adequate statistical power.

Attrition analyses suggested that participants with missing information had worse mental health, they were more likely to identify as having a sexual orientation of gay or “other”, had a smaller family of choice, their family of choice was less stable and committed, they had less close family of origin, their family of origin was less stable and committed, they were less likely to have a partner. They did not differ in terms of age, education, number of chronic illnesses, acceptance, and negativity of their family of choice and family of origin.

## Measures

### Depressive symptoms

Depressive symptomatology was assessed via the center for epidemiological studies depression scale (CES-D) [44]. Participants were asked to rate the frequency of experiencing symptoms “during the past week” on a scale ranging from (1) rarely or none of the time to (4) most or all the time. We used the eight-item scale, shown to have good validity and reliability among older adults [45].

### Well-being

The world health organization (WHO) well-being index is a global rating scale measuring subjective well-being, validated for the older population [46]. The index comprises five statements addressing aspects of the participant’s feelings over the previous two weeks, such as “I felt cheerful and in good spirits.” Responses range over a 6-point Likert scale ranging from (0) at no time to (5) all the time.

### Social relationships

We assessed participants’ social relationships with regard to three categories of people: members of their biological family\family of origin, families of choice, and a partner. We inquired about the characteristics of the relationship with the partner seperately since this is a distinct relationship. Partners are often included in the family of choice because they are chosen relationships rather than biologically determined. However, romantic partnerships involve unique characteristics—such as relationship exclusivity, shared living arrangements, and legal recognition options—that distinguish them from other chosen family bonds. Additionally, the intensity and nature of partner support often differs from that provided by other family of choice members, with partners frequently serving as primary caregivers and confidants. Given these differences, we believe it is important to inquire about them separately. The questions about relationship characteristics were asked in relation to each of these three social categories (e.g., participants were asked about the stability of the relationship with their biological families\families of origin, the stability of the relationship with their families of choice, and the stability of the relationship with their partner).

Questions about the number of ties were assessed by asking how many people are in the respondents “family of choice”, defined as “people with whom you are emotionally close and consider ‘family’ though they are not biologically related to you” [20]. Respondents were also asked how many close biological family members they have. Information about having a partner was gleaned by asking participants to indicate their marital status (single, married, in a civil relationship, in a relationship, divorced\separated, widowed) and those who reported that they are married, in a civil relationship or in a relationship were classified as having a partner. Participants were also asked to indicate, after being asked about the number of family of choice members, who is considered to be in their family of choice. Those who mentioned a partner were also classified as having a partner. If a respondent cited their partner as being part of their family of choice, we subtracted one from the number of family of choice members so that having a partner and number of family of choice members will not overlap.

We assessed relationship stability using two items: “I think it is unlikely that the relationship with my (biological family\family of choice\partner) will end in the future;” “I think my relationship with my (biological family\family of choice\partner) is very stable” [47]. The two items about stability were averaged. Items on commitment were adapted from a commitment scale [48] and included two items: “I feel committed to maintaining the relationship with my biological family\family of choice\partner”; “My biological family members\family of choice members\partner are\is committed to our relationship”. The two items about commitment were averaged. An item regarding sexual identity acceptance inquired: “I am open about my sexual identity with my (biological family\family of choice\partner)” [22]. We also asked about negativity of the relationship by asking: “My (biological family\family of choice\partner) are\is difficult and demanding” [49]. The response options for the items ranged between (1) Not at all to (5) A lot. The responses for family of choice and for partner were averaged to include a fuller sample in the analyses, since some respondents only had a partner without other people in their family of choice, some only had a family of choice but not a partner, while others had both a partner and other people in their family of choice. Thus, examining them separately would have resulted in a partial picture, focusing only on people who have both a partner and a family of choice.

### Covariates

We inquired about different sociodemographic and health aspects known to impact mental health and social relationships in later life [50, 51]. We asked participants about their age, level of education (below high education\high education), sexual orientation (homo\gay,^2^ lesbian, bisexual, heterosexual, other). Participants were also asked to indicate whether they identify on the trans spectrum (yes\no). We included all participants who identified as being on the trans spectrum. Additionally, we also included individuals who identified as “heterosexual” only if they also identified as being on the trans spectrum. In other words, cisgender heterosexual individuals were excluded from the study, while cisgender non-heterosexual individuals and all trans individuals were included. We also inquired about the number of chronic illnesses, ranging from 0 to 7 illnesses.

### Data analysis

First, we employed descriptive statistics and bivariate associations which were examined using Pearson correlations, *t*-tests or Chi-square analyses for continuous, dichotomous, and categorical variables, respectively. We then compared the characteristics of family of choice and families of origin using *t*-tests. Finally, we used linear regression analyses to examine the contribution of different social relationship characteristics to the mental health outcomes.

## Results

Table 1 shows a description of participants’ characteristics, their social relationships and mental health. They were 58 years old on average, they were relatively educated with 77% having higher education. Almost half identified as gay (47%), while 39% identified as lesbian and 9.5% as bisexual, 1% identified as being on the trans spectrum and defining one's sexual orientation as heterosexual and 3% identified as “other”. They had one chronic condition on average.

**Table 1:** Sample characteristics of the study and bivariate analyses

Variable	Mean (SD)	Percentage	Range	Bivariate analyses depressive symptoms	Bivariate analyses well-being
Demographics					
Age	58.45 (7.08)		50–85	*r* = − 0.04	*r* = 0.07
Sexual orientation				*F* = 2.42*	*F* = 0.59
Gay		47.22%			
Lesbian		39.12%			
Bisexual		9.49%			
Heterosexual (trans)		1.39%			
Other		2.78%			
Education (high education)		76.85%		*t* = 4.41***	*t* = − 2.62**
Chronic conditions	1.05 (1.15)		0–7	*r* = 0.17***	*r* = − 0.23***
Mental health					
Depressive symptoms	1.92 (0.63)		1–4		*r* = − 0.76***
Well-being	3.79 (1.01)		1–6	*r* = − 0.76***	
Family of choice					
Number of family of choice	5.12 (4.88)		0–20	*r* = − 0.18***	*r* = 0.19***
Has a partner		67.13%		*t* = 6.00***	*t* = − 5.04***
Stability of the relationship	4.18 (0.76)		2–5	*r* = − 0.32***	*r* = 0.27***
Commitment	4.45 (0.63)		2–5	*r* = − 0.28***	*r* = 0.23***
Acceptance of sexual orientation	4.63 (0.65)		1–5	*r* = − 0.24***	*r* = 0.18***
Negativity	1.72 (0.85)		1–5	*r* = 0.24***	*r* = − 0.20***
Biological\family of origin					
Number of close family of origin	4.15 (3.42)		0–20	*r* = − 0.22***	*r* = 0.25***
Stability of the relationship	4.30 (0.94)		1–5	*r* = − 0.17***	*r* = 0.17***
Commitment	4.34 (0.88)		1–5	*r* = − 0.19***	*r* = 0.17***
Acceptance of sexual orientation	4.20 (1.03)		1–5	*r* = − 0.24***	*r* = 0.16***
Negativity	1.86 (1.05)		1–5	*r* = 0.22***	*r* = − 0.23***

The participants had a high level of well-being (3.8 of 6) but also medium depressive symptoms (1.9 of 4). As for their social relationship characteristics, they reported five members in their family of choice (excluding a partner) and 67% had a partner. They reported high stability and commitment of the relationships with their family of choice (4.2, 4.5 of 5, respectively) and even higher acceptance of their sexual orientation by their family of choice (4.6 of 5), while the negativity of the relationship was relatively low (1.7 of 5). Regarding the relationship with the family of origin, the participants reported high stability and commitment of the relationships with their family of origin members (4.3 of 5) as well as the acceptance of their sexual orientation (4.2 of 5). They reported relatively low negativity of the relationship (1.9 of 5). They were close to four family of origin members on average.

Table 1 also presents the bivariate associations of the study variables with the outcome variables of depressive symptoms and well-being. Having more family of choice members and a partner were associated with better mental health—fewer depressive symptoms and higher well-being. Higher commitment and stability of the relationship, higher acceptance of one's sexual orientation and lower negativity of the family of choice were also linked with better mental health. The characteristics of the relationship with one's family of origin family were also associated with mental health, specifically having more close family of origin members, higher commitment and stability of the relationship, higher acceptance of one's sexual orientation and lower negativity. Age was not associated with mental health, while higher education level and low number of chronic conditions were associated with better mental health. Sexual orientation was associated only with depressive symptoms, although a post-hoc Bonferroni analysis suggested that none of the comparisons differed significantly from each other.

We next conducted *t*-test analyses to compare the relationship characteristics between families of choice and family of origin. These comparisons revealed lower stability of the relationship with the family of choice compared to the family of origin (M (SD) = 4.18 (0.76) vs. M (SD) = 4.30 (0.94), respectively, *t* = 2.35, *p* = 0.019), while there was higher commitment of the relationship with the family of choice compared to the family of origin (M (SD) = 4.45 (0.63) vs. M (SD) = 4.34 (0.88), respectively, *t* = −2.56, *p* = 0.011). The family of choice was more accepting of one's sexual orientation (M (SD) = 4.63 (0.65) vs. M (SD) = 4.20 (1.03), respectively, *t* = − 9.97, *p* < 0.001) and the relationship with them was less negative, compared with the family of origin (M (SD) = 1.72 (0.85) vs. M (SD) = 1.86 (1.05), respectively, *t* = 2.52, *p* = 0.012). We applied the Holm-Bonferroni method to control for false positives in multiple comparisons. Since the p-values remained smaller than their respective adjusted thresholds, they retained statistical significance after this correction.

In the final stage of analysis, we examined the associations of the social relationships of LGBTQ + adults with their mental health, using OLS regression models to predict the two mental health indicators—depressive symptoms and well-being. Table 2 shows the results of these models. The first model predicted depressive symptoms. Fewer depressive symptoms were associated with having more family of choice members, having a partner, rating the relationship with one's family of choice as high in stability and as less negative. Characteristics of the relationship with the family of origin were not related to depressive symptoms. Having higher education and fewer chronic conditions were related to fewer depressive symptoms, while being on the trans spectrum and defining one's sexual orientation as heterosexual was related to more depressive symptoms, compared to being a gay.

**Table 2:** Linear regression models predicting mental health of LGBTQ + adults aged 50 +

	Depressive symptoms			Well-being		
Variable	B	SE	β	B	SE	β
Number of family of choice	− 0.01	0.01	− 0.11*	0.02	0.01	0.10*
Has a partner	− 0.27	0.06	− 0.20***	0.33	0.10	0.15**
Stability—family of choice	− 0.14	0.05	− 0.16**	0.17	0.09	0.13*
Commitment—family of choice	0.03	0.07	0.03	0.02	0.11	0.01
Acceptance of sexual orientation—family of choice	− 0.01	0.05	− 0.01	0.03	0.09	0.02
Negativity—family of choice	0.11	0.04	0.14**	− 0.13	0.06	− 0.11*
Number of close family of origin	− 0.01	0.01	− 0.06	0.03	0.01	0.10*
Stability—family of origin	0.09	0.05	0.13	− 0.10	0.08	− 0.10
Commitment—family of origin	− 0.09	0.05	− 0.13	0.12	0.09	0.10
Acceptance of sexual orientation—family of origin	− 0.04	0.03	− 0.06	− 0.01	0.06	− 0.01
Negativity—family of origin	0.03	0.03	0.06	− 0.08	0.05	− 0.08
Age	− 0.01	0.00	− 0.06	0.01	0.01	0.08
Sexual orientation (lesbian)^1^	0.00	0.06	0.00	0.00	0.10	0.00
Sexual orientation (bisexual)^1^	0.05	0.10	0.02	− 0.06	0.16	− 0.02
Sexual orientation (heterosexual: trans)^1^	0.49	0.24	0.09*	− 0.02	0.40	0.00
Sexual orientation (other)^1^	0.30	0.17	0.08	− 0.10	0.28	− 0.02
Education (high education)	− 0.26	0.07	− 0.17***	0.19	0.11	0.08
Chronic conditions	0.09	0.02	0.16***	− 0.19	0.04	− 0.21***
R^2^			0.29			0.23

B = coefficient, β = standardized coefficient

*p <.05, **p <.01, ***p <.001

1Reference category = Gay

The second model in Table 2 predicted well-being. The number of family of choice members was related to having better well-being, as well as having a partner, high stability and low negativity of the relationship with the family of choice. The number of close family of origin members was related to having better well-being, but other characteristics of the family of origin were not related to well-being. The results also showed significant associations of well-being with having fewer chronic conditions.

## Discussion

The current study set out to examine the characteristics of families of choice and family of origin of LGBTQ + older adults and to explore their associations with mental health. Its findings indicate that families of choice differ from families of origin in several aspects. Namely, the relationship with them is less stable but more committed, they are more accepting of one's sexual orientation and less negative. Families of choice were also found to be more relevant to mental health, in comparison to families of origin. Having a partner and more people in one's family of choice, stability of the relationship and experiencing relationships with families of choice as less negative emerged as associated with mental health. In relation to the family of origin, however, only the number of close family of origin members was related to mental health.

The findings partially confirm the first hypothesis, regarding the comparison between families of choice and families of origin. In accordance with the hypothesis, the relationship with the family of choice was reported to be more accepting of one's sexual orientation and less negative. Moreover, the relationship with the family of choice was described as more committed compared to the family of origin. These findings can indicate that indeed, families of choice offer unique sources of support by being more accepting and less burdensome. This shows the importance of choice, since these close others were likely chosen because of their high acceptance and low negativity. Interestingly, families of choice were also considered to be more committed compared to families of origin (contrasting with our hypothesis). This finding might indicate that members of families of choice will be available to provide care and support in time of need, an issue of high relevance for aging LGBTQ + adults [41, 42]. The higher commitment levels found in families of choice may reflect the intentional nature of chosen family relationships, since these bonds are deliberately cultivated based on mutual understanding and shared experiences, rather than biological obligation [21]. These relationships could be further strengthened by shared experiences of marginalization, which can enhance solidarity and a sense of duty toward one another, and by the possibility of authentic self-expression [16]. However, the family of choice was considered a less stable relationship compared to the family of origin. This lower stability may stem from the perceived resilience of biological ties—encapsulated in the phrase “blood is thicker than water”—which might be (subjectively or objectively) more likely to persist even in less accepting or more negative relationships.

The second study hypothesis concerned the associations between the characteristics of both types of families and mental health. Specific aspects of these unique social relationships, such as level of commitment and stability, were expected to be particularly relevant to the mental health of sexual and gender minorities in later life. This hypothesis was partially confirmed. The stability of the relationship is an aspect of the social ties of LGBTQ + older adults that has not been examined in depth, possibly as it is “taken for granted” in the more prevalent reseach on social relationships in old age, but it is likely a more open question in the lives of LGBTQ + older adults [41, 42]. Having relationships that are stable could mean that someone will provide care when in need, and provide a greater sense of emotional security. Negativity of the relationship was also found to be related to mental health, in accordance with previous research on the heterosexual older population [52]. Thus, even families of choice, which are deliberatly picked as close, can be negative and this can have implications for mental health. The acceptance of one's sexual orientation was less relevant for mental health. Perhaps in later life, these individuals have become used to the level of acceptance from their social environment and thus it has less of an impact compared to other aspects of their relationships.

The number of members in one's family of choice and in the close family of origin was associated with mental health, indicating that it is not only the quality of the relationships but also their mere existence, with more close others potentially being able to offer more support and more varied types of support [38]. Having a partner was particularly relevant. LGBTQ + older adults are less likely to have partners, compared to their heteroxesual countreparts [12]‚ and being able to have such a close relationship provides many benefits [53, 54].

The associations with mental health were seen mostly in the relationships with the family of choice and less in the relationships with the family of origin, corroborating the third study hypothesis. Possibly, families of choice are more central in the lives of LGBTQ + older adults. These findings expand upon previous research, for example a finding that social support from friends, but not from family, was related to lower depression among this group [35]. However, the current results indicate that families of choice in general, not only friends, are important for mental health. This indicates that the outlook on social support resources of aging LGBTQ+ adults should be expanded beyond only those defined as friends to a wider definition.

The finding that only the number of family of origin members was positively related to well-being, while other characteristics were not significant, can be interpreted through several lenses. This pattern suggests that for LGBTQ + older adults, the mere presence of family connections may provide a basic social safety net that offers access to potential sources of support, regardless of relationship quality [55]. Quality aspects such as stability and commitment may be less relevant in this context, possibly due to the complex nature of family of origin relationships for LGBTQ + individuals, who often experience ambivalence or past rejection from family of origin members [14].

The lack of association of mental health outcomes with the stability and acceptance of the family of choice may reflect several underlying processes. This finding could indicate, for example, that negativity and commitment may be more crucial for mental health and that active support and positive engagement matter more than passive qualities such as stability or mere acceptance [56]. The historical context is also relevant: Older LGBTQ + adults may have experienced higher levels of discrimination in earlier life stages, making even moderately accepting relationships feel sufficient, whereas negativity in relationships remains a strong predictor of poor mental health [29].

This study had some limitations that should be acknowledged. A primary challenge when conducting research with the LGBTQ+ community is the ability to obtain a representative sample size from a group that has been historically marginalized, as our sample is likely biased towards older adults who are “out” and publicly acknowledge their sexual identity [1]. To cope with this potential limitation, we used a snowballing method, in addition to actively reaching out through LGBTQ + organizations. We also emphasized the confidentiality of the questionnaires and the importance of the research [12, 32]. The distribution of our survey via online platforms allows us to ensure the anonymity of participants, but it can introduce an additional limitation that our sample is more educated compared to the target population. Hence, we controlled for education level in the multivariate analyses as a partial solution. Another limitation is the cross-sectional nature of the data. Thus, the temporal causal process may differ from our hypothesis and it is possible that people who have better mental health seek out more social ties [57]. Additionally, it should be noted that some respondents were excluded from the final analysis due to missing information, and they differed in certain attributes from those participants who were ultimately included. This pattern of attrition may limit the generalizability of our findings. Despite these limitations, the current study provides a warranted first step in the understanding of social processes and mental health among LGBTQ + adults in later life.

Given the hardships endured by sexual and gender minorities, it is crucial to find ways to improve the well-being of older LGBTQ+ adults and reduce their depressive symptomatology. The current study contributed towards this goal by identifying unique resources for social support. Although the role of social relationships is relatively well understood in the general population, less is known about social relationships regarding the mental health of older LGBTQ + adults. This is even though such knowledge is more important as older LGBTQ + people are usually more dependent on their social networks, as they are often less willing to seek institutional help due to negative experiences in their lives. This study examined a novel theoretical framework that expands the HEPM and the convoy model by delineating the social relationships of sexual and gender minorities in relation to mental health. Particular attention was given to families of choice, which are often a source of support for LGBTQ + older adults, and we delved into unique aspects of these social ties such as their level of stability and acceptance, which are currently not fully understood. The results indicated that aging LGBTQ + adults can have varied sources of social support which could be utilized to better their lives.

These findings have some important implications for practitioners and future research. Mental health professionals should help LGBTQ + older adults develop and strengthen their support networks, with particular attention to families of choice. Interventions could include, for example, facilitating support groups that help LGBTQ + older adults build new connections and strengthen existing ones and provide counseling that addresses concerns about the stability and reliability of chosen family relationships. Practitioners could also assist LGBTQ + older adults in formalizing their family of choice relationships through legal mechanisms like power of attorney, which can help address concerns about commitment and stability. Future research should examine these family of choice relationships through several important lenses. Longitudinal studies are needed to understand how these relationships evolve over time, particularly during life transitions such as health crises. Such studies could also illuminate the bidirectional relationship between mental health and social support networks—investigating whether better mental health leads to stronger family of choice relationships, or if the temporal direction is different. And finally, studies should also examine how intersecting identities (e.g., race, socioeconomic status, gender identity) influence the development and maintenance of chosen family relationships among LGBTQ + older adults.

## Data Availability

This study is not preregistered, the data and materials are not available online because the authors have not completed their original work with the data set. We are happy to share the data upon request.

## Footnotes

1 We use the term “LGBTQ + ” to ensure inclusivity of diverse sexual and gender identities while also reflecting the language commonly used within the studied population. This terminology aligns with contemporary discourse in both academic literature and advocacy organizations relevant to our research context.

2 “Homo/gay” is considered the more acceptable terminology within the Israeli gay community.
